# Exploring flubendazole formulations for use in sheep. Pharmacokinetic evaluation of a cyclodextrin-based solution

**DOI:** 10.1186/1746-6148-8-71

**Published:** 2012-05-28

**Authors:** Laura Ceballos, Laura Moreno, Juan J Torrado, Carlos Lanusse, Luis Alvarez

**Affiliations:** 1 Departamento de Fisiopatología, Laboratorio de Farmacología, Facultad de Ciencias Veterinarias, Universidad Nacional del Centro de la Provincia de Buenos Aires (UNCPBA), Campus Universitario, 7000, Tandil, Argentina; 2 Consejo Nacional de Investigaciones Científicas y Técnicas (CONICET), Tandil, Argentina; 3 Departamento de Farmacia y Tecnología Farmacéutica, Facultad de Farmacia, Universidad Complutense de Madrid, Madrid, Spain

## Abstract

**Background:**

Flubendazole (FLBZ) is a poor water solubility broad-spectrum BZD methylcarbamate anthelmintic compound. Cyclodextrins (CDs) are usually used to increase aqueous solubility of poor hydrosoluble compounds. The comparative *in vitro* aqueous solubility of FLBZ and other BZD anthelmintics in the presence of hydroxypropyl-β-cyclodextrin (HPβCD) was evaluated in the current work. Additionally, the comparative pharmacokinetic behaviour of FLBZ (and its metabolites) administered by the intraruminal (i.r.) or intraabomasal (i.a.) routes to sheep as either an aqueous CDs-based solution or a conventional carboximethylcellulose (CMC) suspension was assessed. Drug solubility studies involving albendazole, mebendazole, oxfendazole and FLBZ were performed in an aqueous solution (pH 1.2 or 7.4) with or without HPβCD (10%, w/v). The pharmacokinetic study involved two experiments. Experiment 1: In a crossover study, sheep received either a FLBZ-CDs solution (n = 3) or a FLBZ-CMC suspension (n = 3) by the i.r. route (3.8 mg/kg). The treatment Groups were reversed after a 21-days washout period. Experiment 2: sheep (n = 4) were treated by the i.a. route with the FLBZ-CDs solution (3.8 mg/kg). Plasma and abomasal fluid samples were collected between 0 and 72 h post-treatment. Samples were analysed by HPLC.

**Results:**

Improvement of FLBZ aqueous solubility due to CDs resulted markedly higher than that observed for mebendazole and albendazole. However, oppositely to what was expected, the absorption-related pharmacokinetic parameters did not show any marked formulation-dependant effect. After the i.a. administration of FLBZ, the AUC and the Tmax of the parent compound were significantly (P < 0.05) reduced, which is consistent with ruminal bypass.

**Conclusion:**

The administration of FLBZ as a CDs-based solution, does not seem to achieve great practical relevance for parasite control in sheep.

## Background

Flubendazole (FLBZ) is a broad-spectrum benzimidazole (BZD) methylcarbamate anthelmintic available for use in human and some domestic animals. It is widely used for parasite control in pigs, chicken, turkeys and game birds. FLBZ is commercially available for oral administration as a paste, tablets, pellet or premix formulations [1]. BZD anthelmintics are extensively biotransformed in all mammalian species studied [2]. FLBZ is metabolized by microsomal and cytosolic fractions obtained from sheep liver and duodenal mucosa into a reduced FLBZ metabolite (R-FLBZ) [3]. R-FLBZ was the main analyte recovered from the bloodstream of FLBZ treated sheep [4], in which only trace amounts of the hydrolysed metabolite (H-FLBZ) were detected.

The antiparasitic activity of BZD anthelmintics largely depends on their affinity for parasite ß-tubulin, the putative mode of action [5], but also on their ability to reach high and sustained concentrations at the site of parasite location; which, in turn, depends on pharmacokinetic, metabolic and tissue distribution processes in the host [6]. Despite the type of helminth involved, the higher the concentrations achieved at the parasite location, the higher the amount of drug reaching the target parasite [7], which is strongly supported by the findings from different *in vivo* studies [8-10] where systemic drug availability and efficacy were simultaneously estimated. Because the low aqueous solubility of the methylcarbamate BZD compounds, poor/erratic gastrointestinal absorption is a common inconvenient for the systemic availability of orally administered BZD in most species [6]. Consequently, enhanced systemic availability of the parent drug/active metabolite obtained by increased drug absorption will correlate with an improved antiparasitic effect.

The cyclodextrins (CDs) are cyclic oligosaccharides which are well known as molecular host capable of including water-insoluble guest molecules via non-covalent interaction within their hydrophobic cavity [11]. The CDs-drug complex formations results on changes in the physicochemical properties of the guest drug. Consequently, enhanced aqueous solubility and bioavailability of guest molecules are common effects observed after the formulation of poorly water soluble drugs with CDs [12]. The ability of CDs to enhance oral absorption of poorly water-soluble drugs has been well documented [13-16]. Furthermore, CDs enhance oral bioavailability of FDA’s Class II compounds (poor aqueous solubility, high permeability) such as the BZD methylcarbamates [17]. In fact, it has been reported that hydroxypropyl-ß-CD (HPßCD) increase the relative bioavailability of albendazole (ABZ) metabolites in sheep [18]. Furthermore, ABZ-CDs complexes enhance the bioavailability of ABZ metabolites in mice, improving its activity against *Trichinella spiralis*[19,20].

Previous work carried out in our laboratory demonstrated that CDs enhanced the absorption and systemic availability of FLBZ in mice [21]. This modified pharmacokinetic behaviour permitted an enhanced drug exposure of the hydatid cysts which increased FLBZ clinical efficacy against cystic echinococcosis developed in mice [21]. While mice infected intraperitoneally with protoescoleces of *E. granulosus* develop cyst in the whole abdominal cavity, sheep naturally infected with *E.granulosus* oncospheres develop hydatid cyst in similar locations (liver, lungs, etc.) to those observed in humans. Complementary work to evaluate the efficacy of FLBZ against cystic echinococcosis developed in sheep is planned. However, before any clinical efficacy trial is performed, the effect of CDs in the pharmacokinetic behavior of FLBZ in sheep needs to be assessed. The comparative *in vitro* aqueous solubility of FLBZ and other closely related BZD anthelmintics in the presence of HPβCD was evaluated in the current work. Based on those solubility results, the comparative plasma and abomasal fluid pharmacokinetic behaviour of FLBZ (and its metabolites) intraruminally (i.r.) administered to sheep as either an aqueous CDs-based solution or a conventional carboxymethylcellulose (CMC) suspension was assessed. Complementary, the FLBZ/metabolites disposition kinetic was characterized after the experimental intraabomasal (true stomach in ruminants) of the FLBZ CDs-based solution.

## Results

The comparative aqueous solubility of different BZD compounds assayed with or without HPßCD at pH 1.2 and 7.4 is shown in Table 1. BZD solubility greatly increased at a pH value of 1.2, in which the highest aqueous solubility (6.6 ± 1.3 mg/mL) was observed for the most polar ABZSO metabolite. Furthermore, the presence of HPßCD significantly enhanced the solubility of the different BZD compounds at both pH values assayed (P < 0.05). At pH 1.2 (with HPßCD), the aqueous solubility resulted ABZSO > FLBZ > OFZ > ABZ > MBZ. However, the greater HPßCD-induced “solubility increasing effect” (pH 1.2) was observed for FLBZ (75 fold), which resulted higher than that observed for OFZ (35 fold), MBZ (20 fold) ABZ (1.3 fold) or ABZSO (0.7 fold) (Table 1).

**Table 1:** Evaluation of the aqueous solubility (μg/mL, n = 6) of different benzimidazole (BZD) compounds with or without hydroxypropyl-ß-cyclodextrin (HPßCD) (10%) at different pHs (1.2 or 7.4)

**BZD compound**	**pH 1.2**		**pH 7.4**	
	**With HPßCD**	**Without HPßCD**	**With HPßCD**	**Without HPßCD**
Albendazole	921.1 ± 347.3	406.1 ± 53.4*	43.0 ± 8.3	4.6 ± 1.3*
Albendazole sulphoxide	6637.7 ± 1370.2	3818.6 ± 202.1*	871.9 ± 129.3	83.6 ± 7.6*
Mebendazole	436.7 ± 47.4	20.5 ± 5.0*	43.0 ± 8.3	4.6 ± 1.3*
Oxfendazole	1902.5 ± 605.8	419.7 ± 29.2*	348.1 ± 46.8	5,5 ± 1.1*
Flubendazole	2251.7 ± 551.5	29.6 ± 2.8*	163.8 ± 119.7	5.8 ± 7.0*

*At each pH value (1.2 or 7.4), significantly different from the solubility obtained in the presence of HPßCD at P < 0.05.

**Figure 1:**
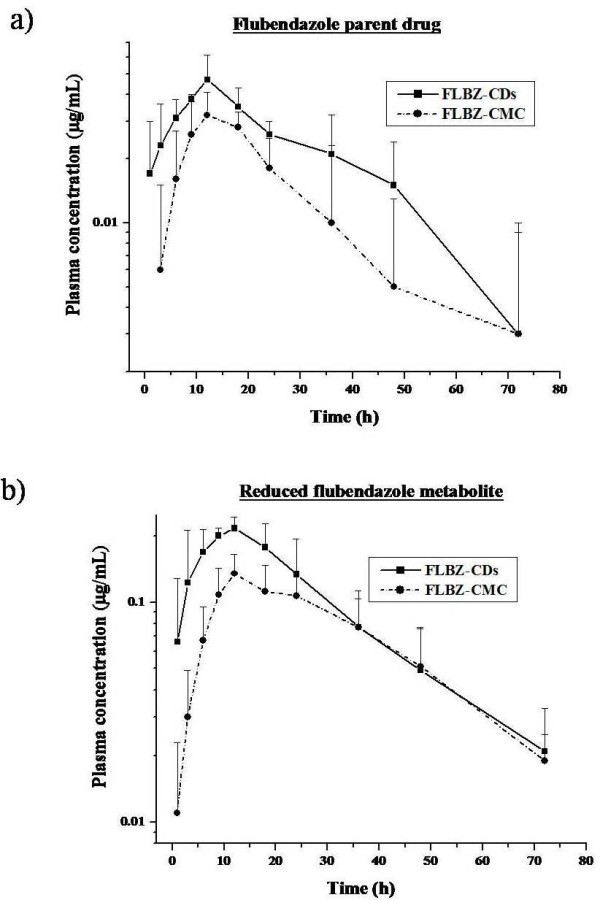
**FLBZ/metabolites plasma concentrations.** Comparative mean (±SD) plasma concentration profiles (n = 6) for **a**) flubendazole (FLBZ), and **b**) reduced flubendazole (R-FLBZ), after the intraruminal (i.r.) administration of FLBZ (3.8 mg/kg) as either, a cyclodextrin (CDs) solution or a carboxymethylcellulose (CMC) suspension to sheep.

The comparative mean plasma concentration profiles of FLBZ (a) and R-FLBZ (b) observed after the i.r. administration of FLBZ formulated both as a CDs-solution and CMC-suspension are shown in Figure 1. In experimental groups (FLBZ-CDs and FLBZ-CMC), the parent drug and its R-FLBZ metabolite were the main analytes recovered in plasma. Low concentrations of the H-FLBZ metabolite were detected in plasma between 18 and 48 h after the administration of FLBZ in all experimental animals, which preclude any pharmacokinetic analysis. The mean pharmacokinetic parameters for FLBZ and R-FLBZ after the administration of FLBZ as a CDs-solution (i.r. and i.a. administration) or CMC-suspension (i.r. administration), are shown in Table 2. FLBZ AUC values were similar between formulations after its i.r. administration. Low peak plasma concentrations for the parent compound were observed after the administration of FLBZ in both CDs-solution (0.05 ± 0.01 μg/mL) and CMC-suspension (0.03 ± 0.01 μg/mL). However, the observed plasma Cmax value for R-FLBZ resulted significantly higher in FLBZ-CDs treated compared to that observed in the FLBZ-CMC treated animals.

**Table 2:** Plasma pharmacokinetic parameters (mean ± SD) for flubendazole (FLBZ) and its reduced metabolite (R-FLBZ), obtained after the intraruminal (i.r.) administration of FLBZ (3.8 mg/kg, n = 6) formulated as a cyclodextrin-based solution (FLBZ-CDs) or a carboximethylcelullose suspension (FLBZ-CMC) to sheep (Experiment 1)

**PHARMACOKINETIC PARAMETERS**	**FLBZ**			**R-FLBZ**		
	**FLBZ-CDs**^**(1)**^**i.r. treatment**	**FLBZ-CMC**^**(1)**^**i.r. treatment**	**FLBZ-CDs**^**(2)**^**i.a. treatment**	**FLBZ-CDs**^**(1)**^**i.r. treatment**	**FLBZ-CMC**^**(1)**^**i.r. treatment**	**FLBZ-CDs**^**(2)**^**i.a. treatment**
T½abs/for (h)	3.67 ± 1.57	2.92 ± 0.94	0.40 ± 1.43*	3.00 ±1.21	4.95 ± 1.72	0.90 ± 0.30*
Cmax (μg/mL)	0.05 ± 0.01	0.03 ± 0.01	0.07 ± 0.04*	0.23 ± 0.04	0.14 ± 0.03*	0.35 ± 0.09*
Tmax (h)	12.0 ± 3.79	12.5 ± 2.95	2.75 ± 2.36*	10.5 ± 4.93	11.5 ± 1.22	2.75 ± 1.50*
AUC_0-t_ (μg.h/mL)	1.35 ± 0.34	0.78 ± 0.53	0.65 ± 0.29	6.82 ± 1.77	4.92 ± 1.46	3.83 ± 1.86*
T½el (h)	25.8 ± 14.0	19.2 ± 15.1	15.1 ± 8.27*	17.0 ± 5.19	18.4 ± 3.74	6.73 ± 3.70*
MRT (h)	41.6 ± 19.0	34.5 ± 20.3	19.7 ± 9.73*	28.8 ± 8.58	35.4 ± 3.90	10.8 ± 4.07*

The pharmacokinetic parameters obtained after the intra-abomasal (i.a.) administration of FLBZ (3.8 mg/kg, n = 4) formulated as a cyclodextrin-based solution (FLBZ-CDs), is also shown (Experiment 2). **T½abs/for**: FLBZ absorption or metabolite formation half life; **Cmax**: peak plasma concentration; **Tmax**: time to the Cmax; **AUC**_**0-t**_: Area under the plasma concentration vs. time curve from 0 to the detection time; **T½el**: elimination half-life; **MRT**: mean residence time (obtained by non-compartmental analysis of the data). *Significantly different from the FLBZ-CDs i.r. treated group at P < 0.05.

^1^Experiment 1: crossover design (n = 6) sheep were treated with the HPβCD-FLBZ solution (FLBZ-CDs) or the CMC-FLBZ suspension (FLBZ-CMC) by the i.r. route at the same dose rate (3.8 mg/kg).

^2^Experiment 2: intraabomasal (i.a.) administration of the FLBZ-CDs solution (3.8 mg/kg, n = 4).

**Figure 2:**
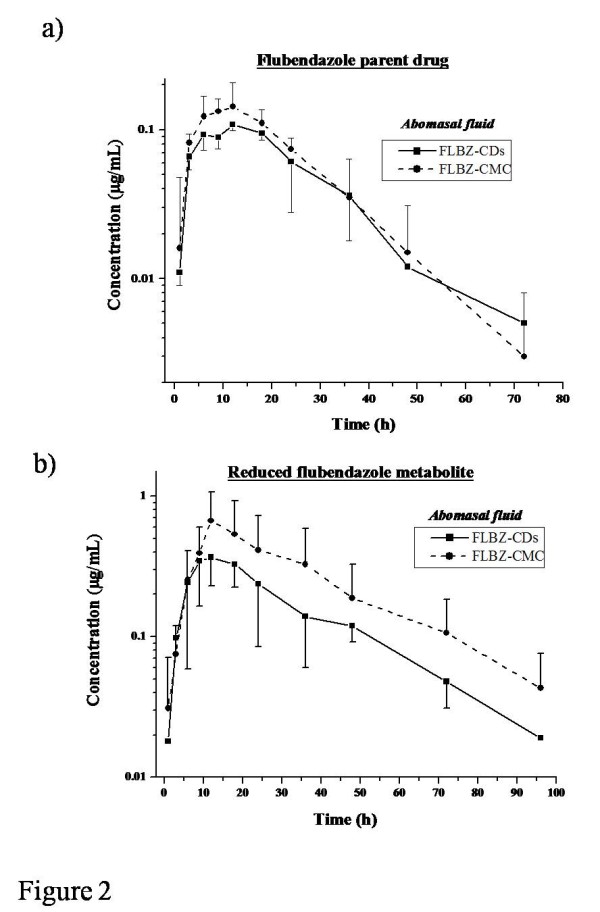
**FLBZ/metabolites abomasal concentrations.** Comparative mean (±SD) abomasal fluid concentration profiles (n = 4) for **a**) flubendazole (FLBZ), and **b**) reduced flubendazole (R-FLBZ), after the intraruminal (i.r.) administration of FLBZ (3.8 mg/kg) as either, a cyclodextrin (CDs) solution or a carboxymethylcellulose (CMC) suspension to sheep.

Figure 2 shows the mean concentration profiles for FLBZ (a) and R-FLBZ (b) in abomasal fluid, obtained after the i.r. administration of FLBZ as both formulations to sheep. The results of the kinetic analysis for these molecules in both experimental groups are summarized in Table 3. FLBZ and R-FLBZ were recovered in abomasal fluid after FLBZ administration as both formulations. Whereas the parent compound was found between 1 and 72 h, the detection of R-FLBZ in abomasal fluid lasted up to 96 h post-treatment. The overall abomasal pharmacokinetic pattern for FLBZ and R-FLBZ was similar in both experimental groups, however a statistically difference in Cmax value (P < 0.05) was observed for R-FLBZ.

**Table 3:** Abomasal fluid pharmacokinetic parameters (mean ± SD, n = 4) for flubendazole (FLBZ) and its reduced metabolite (R-FLBZ) obtained after the intraruminal (i.r.) administration of FLBZ (3.8 mg/kg) formulated as a cyclodextrin-based solution (FLBZ-CDs) or a carboximethylcelullose suspension (FLBZ-CMC) to sheep (Experiment 1)

**PHARMACOKINETIC PARAMETERS**	**FLBZ**		**R-FLBZ**	
	**FLBZ-CDs i.r. treatment**	**FLBZ-CMC i.r. treatment**	**FLBZ-CDs i.r. treatment**	**FLBZ-CMC i.r. treatment**
Cmax (μg/mL)	0.11 ± 0.01	0.16 ± 0.06	0.43 ± 0.13	0.67 ± 0.40*
Tmax (h)	10.5 ± 3.00	9.75 ± 2.87	12.8 ± 3.77	12.0 ± 0.00
AUC_0-t_ (μg.h/mL)	2.95 ± 0.66	3.63 ± 1.05	12.0 ± 5.01	21.7 ± 15.8
T½el (h)	12.3 ± 5.99	9.83 ± 4.23	16.2 ± 7.54	16.3 ± 5.95
MRT (h)	23.0 ± 8.28	20.4 ± 5.23	30.0 ± 12.1	31.2 ± 8.80

**T½abs/for**: FLBZ absorption or metabolite formation half life; **Cmax**: peak plasma concentration; **Tmax**: time to the Cmax; **AUC**_**0-t**_: Area under the plasma concentration vs. time curve from 0 to the detection time; **T½el**: elimination half-life; **MRT**: mean residence time (obtained by non-compartmental analysis of the data). *Significantly different from the FLBZ-CDs i.r. treated group at P < 0.05.

**Figure 3:**
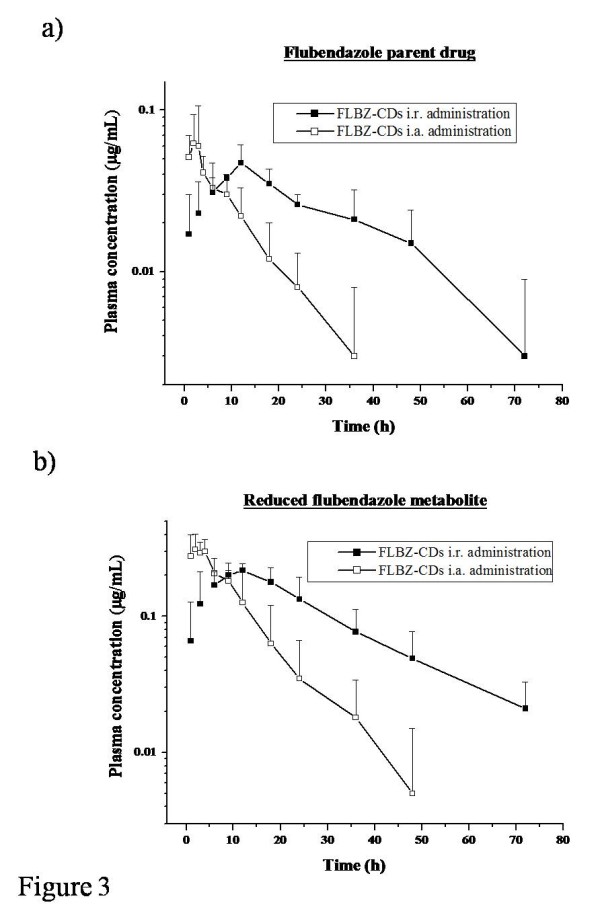
**intraruminal vs intraabomasal plasma concentrations.** Comparative mean (±SD) plasma concentration profiles (n = 4) for **a**) flubendazole (FLBZ), and **b**) reduced flubendazole (R-FLBZ), after the intraruminal (i.r.) or intraabomasal (i.a.) administration of FLBZ (3.8 mg/kg) as a cyclodextrin (CDs) solution to sheep.

The comparative plasma disposition kinetics of FLBZ (a) and R-FLBZ (b) observed after the i.r. and i.a. administration of the CDs-solution are shown in Figure 3. R-FLBZ reached a peak concentration significantly earlier after the i.a. administration (2.75 ± 1.50 h) compared to the i.r. treatment (10.5 ± 4.93 h). On the other hand, the peak plasma concentration of the reduced metabolite resulted higher (P < 0.05) after the i.a. administration (0.35 ± 0.09 μg/mL), compared to that observed after the i.r. treatment (0.23 ± 0.04 μg/mL). However, a faster plasma depletion either for FLBZ or R-FLBZ metabolite was observed in sheep treated by the i.a. route, this represented in both cases a lower AUC value (P < 0.05) compared to that observed after the i.r. FLBZ-CDs administration (1.35 ± 0.34 μg.h/mL and 6.82 ± 1.77 μg.h/mL for FLBZ and R-FLBZ, respectively).

## Discussion

BZD methylcarbamate compounds are insoluble or slightly soluble in water. Solubility studies involving ABZ [22] or ABZSO [23] indicate that BZD methylcarbamate compounds possesses both acidic and basic groups in their chemical structure, with pKa values ranging between 7.2-10.7 and 2.4-4.7, respectively. Thus, the aqueous solubility of these compounds increases greatly when pH values are above 11 or below 2. The impact of low pH values in improving the aqueous solubility of different BZD compounds has been previously demonstrated [24-26]. In the same direction, higher aqueous solubility of different BZD molecules was observed under our *in vitro* experimental conditions (Table 1). A markedly higher aqueous solubility at both, pH 1.2 or 7.4, was obtained for ABZ, ABZSO, MBZ, OFZ and FLBZ. However, the highest solubility improvement was observed for FLBZ at pH 1.2, where the presence of HPßCD enhanced 70 fold its water solubility (Table 1).

FLBZ, R-FLBZ and H-FLBZ were recovered in plasma samples after i.r. administration of FLBZ formulated as either the CDs-solution or CMC-suspension. The active anthelmintic molecule after FLBZ treatment is the parent drug, which exhibits a high affinity binding for ß-tubulin [5] and high potency in parasite motility test [27]. Additionally, the ovicidal activity against *Fasciola hepatica* eggs of the R-FLBZ metabolite has recently been described [28] as well as the *in vitro* activity against *Echinococcus granulosus* protoscoleces [29]. Additionally, this metabolite has demonstrated ability to accumulate (under *ex vivo* conditions) into target parasites equivalent to that observed for the parent FLBZ compound [4]. Consequently, a complementary effect of the parent drug and the reduced metabolite may account for the final anthelmintic activity after FLBZ administration.

The limited aqueous solubility of the most potent BZD compounds allows their formulation only as suspensions for oral administration to sheep. Water solubility and the rate of dissolution in the gastrointestinal (GI) tract lumen have been shown to be relevant to the absorption and resultant systemic availability of BZD compounds in ruminants [6]. To increase the bioavailability of poorly soluble compounds, pharmaceutical scientists may rely on particle micronization, use of surfactant agents, or use of complexing agents such as CDs [7]. Increased ABZ aqueous solubility induced by HPßCD has previously been reported [30]. Moreover, the administration of an ABZ-HPßCD formulation to sheep increased GI absorption of ABZ with an increment (37%) in the AUC value of the active ABZSO metabolite [18]. However, this formulation-based advantage in water solubility did not reach, under our experimental conditions, a beneficial pharmacokinetic result compared to the treatment with the FLBZ suspension. Contrarily to what was expected, the absorption-related pharmacokinetic parameters did not show any marked formulation-dependant effect. Only the Cmax value for the R-FLBZ metabolite was higher (P < 0.05) after administration of the novel CD-based preparation (0.23 ± 0.04 μg/mL) compared to that observed after the treatment with the CMC-suspension (0.14 ± 0.03 μg/mL) (Table 2). The AUC values obtained after the administration of a CDs-based formulation resulted 73% (FLBZ) and 39% (R-FLBZ) higher than those obtained for the CMC-based formulation. However, these differences did not reach statistical differences due to the large variation among experimental animals.

In mice treated with the FLBZ-HPßCD solution, the enhanced FLBZ absorption resulted in a significantly higher (>450%) plasma Cmax compared to that obtained after the FLBZ-suspension treatment (P < 0.05) [21]. This modified pharmacokinetic behaviour permitted a higher drug exposure of the hydatid cysts developed in mice, which enhanced the clinical efficacy of FLBZ in echinococcosis [21]. The drastic changes induced by CDs in the systemic availability of FLBZ in mice, do not correlate with the results observed in sheep. Chemical stability of numerous foreign compounds is affected by the microflora in the rumen (the first forestomach in ruminants). CDs are extensively metabolized in the human colon [15]. It is then likely that after the i.r. administration of the CD-FLBZ complex, a ruminal microflora-mediated metabolic process may have hydrolized/destroyed the CDs structure. This may facilitate FLBZ release into the ruminal fluid, which could then be associated to the particulate ruminal material as it occurs after the treatment with the suspension formulation. Such a phenomenon may account to explain the equivalent FLBZ systemic availability obtained following administration of two pharmaceutically well distinguish formulations. The absence of significant kinetic changes observed for FLBZ/metabolites in abomasal fluid after its administration as a CDs or CMC-formulation supports this argument.

To confirm the hypothesis of a CDs ruminal degradation, a pharmacokinetic study involving the i.a. administration of FLBZ as a CDs-based solution was performed. The plasma disposition kinetics of FLBZ/metabolites after its i.a. administration agrees with those reported after the i.a. administration of FBZ [31], OFZ [32] and ABZ [33] in sheep. Basically, the AUC and the Tmax of the parent compound were significantly (P < 0.05) reduced by ruminal bypass. Furthermore, a significantly (P < 0.05) shorter T½abs for FLBZ compared to the i.r. administration was observed (Table 2). Similar pharmacokinetic differences were observed for the R-FLBZ metabolite. When an orally administered BZD suspension reaches the rumen, an extensive adsorption of BZD molecules to the rumen particulate digesta has been shown to occur shortly after treatment [21]. This extensive association between drug molecules and the particulate material of the digesta does not inhibit absorption but delays the rate of passage of the drug down the GI tract. The rumen acts as a “drug reservoir” and prolongs the duration of drug absorption [21]. After the direct i.a. administration of a conventional BZD suspension, the drug formulation may bypass the rumen and the advantage of the “ruminal reservoir effect” is lost, resulting in poor dissolution of the BZD suspension in the abomasum due to a shorter residence time at the acidic abomasal pH, which results in reduced intestinal absorption and lower systemic availability. After the i.a. administration of FLBZ as a HPßCD-based solution, we expected to observe an enhanced absorption and subsequent bioavailability of the parent compound. However, the CDs formulation behaved similar to the conventional suspension. The association (adsorption) of FLBZ with digestion particulate material could explain the lack of a clear effect on drug absorption induced by the novel formulation (FLBZ-CDs solution).

## Conclusions

In conclusion the enhanced systemic availability FLBZ induced by CDs, did not reach a significant pharmacokinetic improvement, as it has been shown for monogastric species and does not seem to achieve a great practical relevance for use in parasite control in sheep. The work described here contributes with relevant information on the search of new and/or alternative pharmaceutical strategies to improve the systemic availability of well know anthelmintic drugs for use in ruminant species.

## Methods

### Chemicals

Pure reference standards of FLBZ, reduced-FLBZ (R-FLBZ) and hydrolyzed-FLBZ (H-FLBZ) were kindly provided by Janssen Animal Health (Beerse, Belgium). Reference standards of oxibendazole (OBZ, used as internal standard), ABZ, albendazole sulphoxide (ABZSO) and mebendazole (MBZ) were obtained from Schering Plough (Kenilworth, NJ, USA), and oxfendazole (OFZ) from Rhone Merieux (Lyon, France). The solvents HPLC grade (acetonitrile and methanol) and Potassium phosphate (HPLC grade) were from Sintorgan S.A. (Buenos Aires, Argentina) and J.T. Baker (Phillipsburg, NJ, USA), respectively. Cargill Inc. (Hammond, IN, USA) kindly supplied us the HPßCD. CMC was purchased from Anedra (Buenos Aires, Argentina).

### In vitro dissolution study

BZD solubilities were determined in aqueous solutions at pH values of 1.2 (HCl/KCl) and 7.4 (KH_2_PO_4_) with or without HPßCD (10%). An excess amount of ABZ, ABZSO, MBZ, OFZ or FLBZ was suspended in glass tubes containing 10 mL of each medium. The tubes were placed in a water bath at 25°C with a constant shaken rate of 100 strokes/min for two weeks (Yamato BT-31, Yamato Scientific Co., Japan). Following equilibration, samples were centrifuged at 3000 x g for 10 min (IEC CL 30R, Thermo Electro, Corporation, USA). The supernatants were collected and filtered through a 0.2 μm filter (Whatman Inc., NJ, USA). After filtration, samples were analyzed for drug quantification by HPLC following the method described below.

### In vivo pharmacokinetic study

#### FLBZ formulations

FLBZ-solution (2% w/v) was prepared by dissolution of 2 g of pure FLBZ and 10 g of HPßCD in 100 mL of dionized water (pH 1.2). The pH was adjusted using hydrochloric acid (25 mM). The formulation was shaken during 48 h (40°C) and then was filtrated through a 0.45 μm filter (Whatman, NJ, USA). The final FLBZ concentration was confirmed by HPLC (n = 4). The FLBZ suspension (2% w/v) was performed by addition of 2 g FLBZ pure standard in a suspension of CMC (0.5% w/v) prepared with deionized water (100 mL, pH = 6.0) under shaking (24 h). The FLBZ suspension was vigorously shaken before its administration to sheep.

#### Experimental animals

Six (6) parasite-free Corriedale male sheep (average weight: 50.6 ± 6.0 kg) were used in this experiment. Animals were kept indoors with commercial balanced food and water *ad libitum* for two months prior and during the trial. Four (4) animals were surgically fitted with a permanent cannula in the pyloric region of the abomasum following an adaptation of the technique described by Komarek [34]. A 6-week post-surgery recovery period was allowed before starting the pharmacokinetic trials. Animal procedures and management protocols were approved by the Ethics Committee according to the Animal Welfare Policy (act 087/02) of the Faculty of Veterinary Medicine, Universidad Nacional del Centro de la Provincia de Buenos Aires (UNCPBA), Tandil, Argentina *(*http://www.vet.unicen.edu.ar*).*

### Experimental design, treatments and sampling

#### Experiment 1: Intraruminal treatment

The experiment was conducted following a crossover design with two different experimental phases. In phase I, sheep were treated with the HPβCD-FLBZ solution (FLBZ-CDs Group, n = 3) or the CMC-FLBZ suspension (FLBZ-CMC Group, n = 3) by the i.r. route at the same dose rate (3.8 mg/kg). Intraruminal administration was accomplished by injecting the FLBZ formulation (CDs- or CMC-based) into the rumen through the body wall of the left paralumbar fossa using a 16Gx1½ inch needle. Two animals in each group had a cannula fitted in the abomasum. The treatment groups were reversed after a 21-days washout period. Blood and abomasal fluid samples were collected prior to treatment and at 1, 3, 6, 9, 12, 18, 24, 36, 48, 72 and 96 h post-treatment. Immediately after collection, plasma was separated by centrifugation at 3000 x g for 15 minutes. Plasma and abomasal fluid samples were placed into plastic vial and stored at −20°C until analyzed.

#### Experiment 2: Intraabomasal treatment

Based on the results obtained after the i.r. treatment, a complementary experiment with intraabomasal (i.a.) administration of the FLBZ-CDs solution was performed. The i.a. administration of the HPβCD-FLBZ solution was done by direct injection of the formulation through the intra-abomasal cannulae avoiding liquid losses, at 3.8 mg/kg. Blood samples were obtained prior to treatment and at 1, 2, 3, 4, 6, 9, 12, 18, 24, 36, 48 and 72 h post-treatment. The recovered plasma was stored at −20°C until analyzed.

#### Plasma samples extraction

Plasma and abomasal fluid samples (1 mL) were spiked with 20 μL of OBZ (stock solution 50 μg/mL) as internal standard. FLBZ and its metabolites were extracted using disposable cartridges (Strata®, Phenomenex, CA, USA) previously conditioned with 0.5 mL of methanol, followed by 0.5 mL of water, as previously described by Moreno et al. [4]. All samples were injected into cartridges and then sequentially washed with 1.5 mL of water and eluted with 2 mL of methanol. For abomasal fluid samples there was a further extraction step using ethyl acetate before C18 cartridge extraction, as reported by Lanusse et al. [35]. In both cases (plasma and abomsasal fluid samples), the elutant was evaporated to dryness in a vacuum concentrator (Speed-Vac®, Savant, CE), then reconstituted with 200 μL of the same mobile phase used on the HPLC system. Additionally, a calibration curve was performed by mean of spiked plasma/abomasal fluid with known concentrations of pure standards of FLBZ/metabolites (fortified samples). Experimental and fortified samples were analyzed for FLBZ, R-FLBZ and H-FLBZ, and the internal standard by HPLC.

#### HPLC analysis

Experimental and fortified samples of plasma and abomasal fluid were analyzed for FLBZ, R-FLBZ, H-FLBZ and the internal standard (OBZ) by HPLC following the method described by Moreno et al. [4]. Briefly, 50 μL of sample were injected in a Shimadzu 10 A HPLC System (Kyoto, Japan), using an UV detector set at 292 nm, an autosampler and a controller (Shimadzu Class LC10, Kyoto, Japan). Elution from the stationary phase (Selectosil C_18_ column, 5 μm, 250 × 4.6 mm, Phenomenex®, CA, USA) has been carried out at a flow rate of 1.2 mL/min, using a mixture of acetonitrile:potassium phosphate buffer (25 mM, pH 5.3) (40:60) as a mobile phase, during 13 min at 30°C.

The calibration curves for each analyte constructed by least squares linear regression analysis, showed good linearity with correlation coefficients greater than 0.993. Recovery of the three molecules under study was estimated by comparison of the peak areas from spiked plasma/abomasal fluid, resulting from direct injections of standards in mobile phase. The absolute recovery for FLBZ, R-FLBZ and H-FLBZ ranged between 73 and 94% with coefficients of variation (CV) ≤ 9%. The limit of quantification (LOQ) was defined as the lowest measured concentration with a percentages of residual standard deviation (%RSD) <20%, an accuracy of ± 20% (measured as percentage of relative error) and an absolute recovery ≥70%. The LOQ obtained for the assayed molecules was 0.01 (plasma) and 0.05 (abomasal fluid) μg/mL. Values below LOQ were not included in the pharmacokinetic analysis.

#### Pharmacokinetic analysis of the data

The concentration vs time curves for FLBZ and/or its metabolites in plasma for each individual animal were fitted with the PKSolutions^TM^ computer program (Summit Research Service, OH, USA). The following equation [36] was used to describe the biexponential concentration–time curves for FLBZ and R-FLBZ after the i.r./i.a treatments:

(1) $$Cp=Be^{-\beta t}-Be^{-kt}$$

where Cp = concentration (μg/mL) in plasma at time *t* after administration; B = concentration at time zero extrapolated from the elimination phase (μg/mL); *e* = base of the natural logarithm; *β* = terminal slope (/h); and *k* is the slope determined by feathering which represents either the first-order absorption rate constant (*k*_*ab*_) or first-order metabolite formation rate constant (*k*_*fpr*_) (/h). The elimination half life (T½el) and absorption (T½ab) or metabolite formation (T½for) half lives were calculated as ln2/*β* and ln 2/*k*, respectively. The peak concentration (Cmax) and time to peak concentration (Tmax) were read from the plotted concentration–time curve of each analyte. The area under the concentration–time curve (AUC) was calculated by the trapezoidal rule [37]. Statistical moment theory was applied to calculate the mean residence time of FLBZ and R-FLBZ in plasma and abomasal fluid samples as follow:

(2) MRY=AUMC/AUC

where AUC is defined previously and AUMC is the area under the curve of the product of time and the plasma drug concentration vs time from 0 to ∞ [37].

#### Statistical analysis of the data

The *in vitro* aqueous solubility values are presented as arithmetic mean ± SD. Student´s t-test was used to compare BZD water solubility values, obtained with or without HPßCD, and at two different pHs (1.2 or 7.4). The pharmacokinetic parameters and concentration data are also reported as arithmetic mean ± SD. Statistical comparison of mean pharmacokinetic parameters for FLBZ/metabolites administered by the i.r. route as a HPßCD-based solution or suspension (Experiment 1) was performed using Student´s t-test for paired observations (plasma samples) or Mann–Whitney non parametric test (abomasal fluid samples). The comparison of the plasma and abomasal fluid pharmacokinetic parameters obtained for FLBZ/metabolites after the i.r. or i.a. (Experiment 2) administration was performed by non parametric test (Mann–Whitney test). In all cases, a value of P < 0.05 was considered statistically significant. The statistical analysis was performed using the Instat 3.0 Software (Graph Pad Software, CA, USA).

## Competing interests

The authors declare that they have no competing interests.

## Authors' contributions

LC, LM and LA participate in the animal and analytical phase of the *in vivo* experiments and in writing the draft manuscript. JT carried out the *in vitro* experiment. LA and CL conceived the study, participated in its design and in the animal phase, and revised the draft version of the manuscript. All authors have read and approved the final manuscript.
